# Prevalence and associated factors of symptoms of pica among adolescent schoolchildren in northern Sudan: a cross-sectional study

**DOI:** 10.1186/s40337-023-00777-0

**Published:** 2023-03-27

**Authors:** Mohamed A. Ahmed, Abdullah Al-Nafeesah, Ashwaq AlEed, Ishag Adam

**Affiliations:** 1 Faculty of Medicine, University of Khartoum, Khartoum, Sudan; 2 Department of Pediatrics, Unaizah College of Medicine and Medical Sciences, Qassim University, Unaizah, 56219 Saudi Arabia; 3 Department of Pediatrics, College of Medicine, Qassim University, Buraydah, Saudi Arabia; 4 Department of Obstetrics and Gynecology, Unaizah College of Medicine and Medical Sciences, Qassim University, Unaizah, 56219 Saudi Arabia

**Keywords:** Geophagy, Pica, Adolescent, Associated factors, Sudan

## Abstract

**Background:**

Pica, the craving for and purposive eating of non-food items, is a common worldwide problem, especially among children and pregnant women. There are few published data on pica among adolescents in sub-Saharan Africa, and no study has been carried- out in Sudan. This study was conducted to determine the prevalence and associated factors of symptoms of pica among adolescent schoolchildren in northern Sudan.

**Methods:**

A school‑based cross‑sectional study was conducted from July to September 2022 among adolescent students (aged 10–19 years) in four public primary and secondary schools in Almatamah locality in north Sudan. Sociodemographic information (age, sex, mother’s education, mother’s occupation, and father’s education) was collected through a questionnaire. Weight and height were measured using a standard procedure, and the body mass index Z-score was computed using the World Health Organization’s reference values. Logistic regression analysis was performed.

**Results:**

Of the 384 enrolled adolescents, 180 (46.9%) were male and 204 (53.1%) were female. Their median (interquartile range) age was 15.1 (13.1‒16.3) years. The prevalence of symptoms of pica was found to be 30.7%, accounting for 118 adolescents. The most common type of pica was geophagia (eating clay and sand; 102 adolescents, 86.4%), followed by pagophagia (eating ice; 14 adolescents, 11.8%) and flour (starch; two adolescents, 1.6%). In the logistic regression analysis, females (adjusted odds ratio = 3.52, 95% confidence interval (CI) = 2.15‒5.78) and a lower level of father’s education (adjusted odds ratio = 2.05, 95% CI = 1.26‒3.34) were associated with symptoms of pica.

**Conclusion:**

In Sudan, symptoms of pica are common among adolescents, especially females. Caregivers need to assess pica in adolescents. Further research is needed to develop guidelines, medical training, and practice.

## Abstract (summary)

We conducted this study from July to September 2022 to estimate the rate of adolescents who would eat non-food items (pica) in Sudan. We gathered sociodemographic characteristics through a questionnaire. Of the 384 enrolled adolescents, 180 (46.9%) were male and 204 (53.1%) were female. We found that 118 (30.7%) adolescents would eat non-food items (pica) and that the most common type was eating clay and sand (102 adolescents, 86.4%), followed by eating ice (14 adolescents, 11.8%) and flour (starch; 2 adolescents, 1.6%). Females and those with a lower level of father’s education were likely to eat non-food items.

## Background

Pica is a new diagnostic entity in the fifth edition of the Diagnostic and Statistical Manual of Mental Disorders (DSM-5; American Psychiatric Association) [1] and in the 11th version of the International Classification of Diseases (World Health Organization) [2].

Geophagy (“consumption of earth”), amylophagy (“consumption of raw starches such as cornstarch or uncooked rice”), and pagophagy (“consumption of large quantities of ice”) are the most common types of pica [3]. Pica, the craving and purposive eating of non-food items, is a common worldwide problem, especially among children, adolescents, and pregnant women [4–8]. Recent reports have shown that not only pregnant women have pica but also non-pregnant women and men [9]. Pica and geophagia are associated with anemia [5, 10], gastrointestinal obstruction, and heavy metal poisoning [11, 12]. If picked up in childhood, pica can continue into adulthood and remain throughout life [9]. Several co-morbidities, such as symptoms of avoidant/restrictive food intake disorder, general eating disorders, depression, and anxiety, and less positive body image have recently been reported to be associated with pica [13]. The prevalence of pica among children has been reported to vary between countries [6–8, 14–17]. Several factors, such as sex (female) and family history of pica, have been associated with the disorder [14]. Craving, alleviating gastrointestinal upset, hypersalivation, religious practice, culture, being a natural stimulant, and feeling relief are the main reasons for pica [9, 18, 19].

Adolescents are young, growing individuals [20]. It has been estimated that there are 1.2 billion adolescents aged between 10 and 19 years in the world, 90% of whom are in low- or middle-income countries [21]. There are specific health programs for adolescents in several countries, although this is not the case for those in Sudan, which is the second-largest African country. Adolescents in Sudan have recently been shown to have a high prevalence of obesogenic behaviors and bullying victimization [22]. Sudanese adolescents have been found to have a high level of anemia and trace element deficiency [23]. Pica is widely practiced by Sudanese women [24], and it is associated with anemia in pregnant women [25] and schoolchildren in Sudan [26]. Thus, the current study was conducted to investigate the prevalence and factors associated with pica among adolescent schoolchildren in Sudan. The results of this study will be useful for caregivers, researchers, and health planners to assess the magnitude of the problem and to provide evidence-based interventions.

## Methods

### Study area

River Nile is one of the 18 states of Sudan. Based on the 2008 census, its total population was 1,120,441 [27]. The state has seven localities (the lowest administrative units in Sudan).

### Study population and design

A school‑based cross‑sectional study was conducted from July to September 2022 among students aged 10–19 years in different public primary and secondary schools in Almatamah locality. Almatamah locality is adjacent to Khartoum state, about 120 km from Khartoum, the capital of Sudan. Prior permission was obtained from the Director of School Education, the Government of Almatamah, and the selected school administrators to conduct the study. Informed written consent was signed by the students’ parents (guardians). Four primary and secondary public schools (i.e., Hajer Alteer, Athawra Kabota, Alkoumer, and Wadi Alshohda) were chosen from the 16 schools in the locality’s randomly selected schools using a systematic sampling method. A total of 4,931 students were registered in these four schools. A list of primary- and secondary‑level public schools was collected from the Directorate of Education in Almatamah. From the identified schools, subjects were randomly selected from student class lists based on a systematic random sampling approach. The total sample size (384) was randomly selected from the four schools through the proportional allocation of samples to the total number of students in each school. The total samples of each school were again distributed proportionally to each grade (literacy level). Finally, students in each grade were selected using systematic random sampling methods. The consenting students were interviewed using a questionnaire, which was filled in by four trained medical officers (two males and two females), and their confidentiality was maintained. The questionnaire contained information regarding sociodemographics (age, sex, mother’s education, mother’s occupation, and father’s education). In this study, we followed the definition of symptoms of pica as the “persistent desire to eat uncooked food or non-food substances for at least one month,” which was recently used in Uganda. Moreover, the adolescents were asked questions modified from those asked of pregnant women in Sudan and Uganda: “*What type of uncooked or non-food substance do you desire or eat?*” “*Have you eaten this substance during the last month?*” and “*If the answer to the above question is yes, what type of non-food substance do you crave or consume?*” Thereafter, each participant’s weight and height were taken twice, and the average was calculated using standard procedures. The body mass index (BMI) Z-score was computed using the World Health Organization’s BMI Z-score reference [28].

### Sample size

The sample size of 384 adolescents was estimated (n), with an assumed maximum (50%) prevalence of symptoms of pica among adolescents. A maximum prevalence (50%) [29] of symptoms of pica was assumed because there were no data on the prevalence of symptoms of pica in children in Sudan. This was calculated using a single proportional formula (*n* = Z^2^pq/d^2^). Q = (1 − *p*), Z1 − α = confidence interval (CI) of 95% = 1.96, d = margin of error of 5% = 0.05[30].

### Statistics

Data were entered into the computer using the IBM Statistical Package for the Social Sciences® for Windows version 22.0 (SPSS Inc., New York, USA). The proportions were expressed as frequencies (%). Continuous data were evaluated for normality using the Shapiro–Wilk test and were non-normally distributed. The non-normally distributed data were expressed as median (interquartile range (IQR)). Univariate analysis was performed with symptoms of pica as the dependent variable and sociodemographics (age, gender, BMI Z-score, and educational level) as the independent variables. Variables with *P* < 0.20 in the univariate analysis were entered in a logistic regression with backward elimination. The adjusted odds ratios (AORs) and 95% CI were calculated. A two-sided *P*-value of < 0.05 was considered statistically significant.

## Results

Of the 384 enrolled adolescents, 180 (46.9%) and 204 (53.1%) were male and female, respectively. The median (IQR) of their age and BMI Z-score was 15.1 (13.1‒16.3) years and − 0.6 (− 1.5‒0.3), respectively. The fathers of 254 (66.1%) and the mothers of 241 (62.8%) enrolled adolescents had an education equivalent to or more than the secondary level. The majority (90.9%) of the mothers were housewives.

The prevalence of symptoms of pica was 30.7%, accounting for 118 adolescents. The most common type of pica was geophagia (eating clay and sand; 102/118 adolescents, 86.4%), followed by pagophagia (eating ice; 14/118 adolescents, 11.8%) and consuming flour (starch; 2/118 adolescents, 1.6%). Compared with males, a significantly higher number of females showed symptoms of pica. There was no significant difference in age, BMI Z-score, father’s education level, mother’s education level, and mother’s occupation among adolescents with symptoms of pica and those without (Table 1). Logistic regression analysis showed that females (AOR = 3.52, 95% CI = 2.15‒5.78) and those with a lower level of father’s education (AOR = 2.05, 95% CI = 1.26‒3.34) were associated with symptoms of pica (Table 2).

**Table 1:** Univariate analysis of the factors associated with pica among adolescents schoolchildren in Sudan, 2022

Variables		Total (number = 384	Adolescents who consumed pica (number = 118)	Adolescents who did not consume pica (number = 266)	OR	95% CI	*P*
*Median (interquartile range)*							
Age, years		15.1 (14.0‒16.3)	15.3 (14.1‒16.7)	15.0 (13.9‒15.9)	1.14	0.99‒1.31	0.056
Body mass z-score		− 0.62 (− 1.5‒0.35)	− 0.41 (− 1.3‒0.50)	− 0.74 (− 1.6‒0.30)	1.11	0.95‒1.30	0.167
*Frequency (proportion)*							
Sex	Male	180 (46.9)	33 (28.0)	147 (55.3)		Reference	
	Female	204 (53.1)	85 (72.0)	119 (44.7)	3.22	2.01‒5.14	< 0.001
Mother education	≥ secondary level	241 (62.8)	70 (59.3)	171 (64.3)		Reference	
	< secondary level	143 (37.2)	48 (40.7)	95 (35.7)	1.24	0.79‒1.93	0.337
Mother occupation	Housewife	349 (90.9)	105 (89.0)	244 (91.7)		Reference	
	Employed	35 (9.1)	13 (11.0)	22 (8.3)	1.33	0.64‒2.72	0.436
Father education	≥ secondary level	254 (66.1)	70 (59.3)	184 (69.2)		Reference	
	< secondary level	130 (33.9)	48 (40.7)	82 (30.8)	1.54	0.98‒2.42	0.058

**Table 2:** Adjusted logistic regression analysis of the factors associated with pica among adolescents schoolchildren in Sudan, 2022

Variables		OR	95% CI	*P*
*Median (interquartile range)*				
Age, years		1.09	0.94‒1.26	0.253
Body mass z-score		1.10	0.93‒1.31	0.231
*Frequency (proportion*				
Sex	Male		Reference	
	Female	3.52	2.15‒5.78	< 0.001
Father education	≥ secondary level		Reference	
	< secondary level	2.05	1.26‒3.34	0.004

## Discussion

The current study showed that the prevalence of symptoms of pica in adolescents in northern Sudan was 30.7%. Geophagia (eating clay and sand; 102/118, 86.4%) and pagophagia (eating ice) were the most common types of pica. Our results reported a 30.4% prevalence of symptoms of pica among adolescents, which is lower than the prevalence rate (74.4%) previously reported among children 7–15 years old in Zambia [14]. Moreover, the prevalence of symptoms of pica in our study was lower than that among primary schoolchildren aged 10–18 years in western Kenya (73.1%) [15]. We previously observed that 160 out of 396 (40.4%) pregnant Sudanese women practiced pica [31]. Recently, Nakiyemba et al. reported that 57.0% of pregnant women in Uganda practiced pica [4].

Conversely, the prevalence of pica in our study was much higher than that reported among children in Ghana (9.1%) [6], Iran (6.7%) [7], Switzerland (10.0%) [8], Germany (12.3%) [16], Nepal (12.5%) [17], and France (0%) [32]. It should be noted that it could be difficult to assess the actual/true prevalence of pica because its definition varies, different methods are used to collect data, and some people may not report practicing pica or even do it secretly [19]. Furthermore, the prevalence of pica may vary in different populations due to different reasons for practicing it, such as religious practice, culture, and famine [18].

In the current study, geophagia was found in 86.4% of adolescents and pagophagia in 11.8%. This is consistent with our previous results, which showed that soil was the most consumed pica substance by pregnant Sudanese women [31]. Likewise, Abu et al. reported clay/soil/dust, paper, and chalk to be the most common materials consumed by children with pica in Ghana [33]. Sadeghzadeh et al. found that soil (62.3%) and paper (31.2%) were the materials most consumed by children in Iran [7]. Eating clay, sand, and ice was the most commonly practiced form by pregnant women in Uganda [4].

Our results showed that females had a 3.5 times higher risk of pica, consistent with previous reports showing that geophagy was more prevalent in girls (80.2%) than in boys 67.7%) in Zambia [14]. Conversely, a previous study showed no significant difference in gender and pica prevalence in Iran [7] and Germany [13]. In the current study, a lower level of father’s education was associated with pica (AOR = 2.05). However, previous studies have revealed that education levels were not associated with pica [13, 34]. The prevalence of pica was the same between women who received education about pica and those who did not [35]. Although our results showed no association between age and pica, previous studies have found age to be associated with pica [36, 37].

This study has some limitations. The validated measure called “pica, avoidant/restrictive food intake disorder, and Rumination Disorder Interview” was not used in the study. Thus, we were not able to clearly trace back to the DSM-5 criteria [1] and exclude other sociocultural influences and mental or medical conditions.

## Conclusion

In Sudan, pica is common among adolescents, especially females. Caregivers should assess pica in adolescents. Further research is needed to develop guidelines, medical training, and practice.

## Data Availability

The datasets generated and/or analyzed during the current study are not publicly available (because the manuscript is still under the peer review process) but are available from the corresponding author upon reasonable request.

## Abbreviations

BMI: Body mass index
BAZ: BMI-for-age Z-score
CI: Confidence interval
SPSS: Statistical package for the social sciences
IQR: Interquartile range
WHO: The world health organization
